# Sweet and Salty: Diabetic Ketoacidosis in a Patient With Nephrogenic Diabetes Insipidus

**DOI:** 10.7759/cureus.12682

**Published:** 2021-01-13

**Authors:** Hong A De Sa, Sunhee Chung, Paul M Shaniuk

**Affiliations:** 1 Internal Medicine-Pediatrics, University Hospitals Cleveland Medical Center/Rainbow Babies and Children’s Hospital, Cleveland, USA; 2 Pediatric Emergency Medicine, Oregon Health & Science University, Portland, USA; 3 Medicine, Louis Stokes Cleveland VA Medical Center/Case Western Reserve University School of Medicine, Cleveland, USA

**Keywords:** diabetic ketoacidosis (dka), nephrogenic diabetes insipidus, adolescent diabetes, diabetes mellitus type 2

## Abstract

The co-existence of nephrogenic diabetes insipidus (NDI) with diabetes mellitus (DM) in a patient that presents in diabetic ketoacidosis (DKA) is rare and, to our knowledge, has not been described even in case reports. We report the case of a 16-year-old male with known NDI who presented to the pediatric emergency department (ED) for one day with generalized weakness and decreased appetite, found to be in moderate DKA from new-onset DM. The initial management of his dehydration and hyperosmolar state presented a unique challenge. Fluid resuscitation with isotonic fluids in a patient with NDI poses a risk of worsening hypernatremia, which can lead to seizures and death. However, the use of hypotonic fluids has the potential to lower serum osmolality too quickly, which can result in cerebral edema. Nephrology, endocrinology, and the pediatric intensive care unit (PICU) consultants were notified of this patient, and a discussion was coordinated between sub-specialists to determine the appropriate fluid resuscitation. The patient was allowed to drink free water in addition to receiving intravenous fluids (IVF) of dextrose 5% with 0.2% sodium chloride at a rate of one-and-a-half maintenance (150 mL/hr) in the ED prior to transfer to the PICU where insulin infusion was initiated. This case report provides guidance to inpatient providers on the management of patients with co-existent NDI and DM in DKA, a rare combination that requires thoughtful and urgent management.

## Introduction

The word "diabetes" comes from a Greek word meaning "siphon," referring to the passing of water [1]. Diabetes insipidus (DI) and diabetes mellitus (DM) share similar namesakes because they are both characterized by excessive thirst (polydipsia) and excessive urination (polyuria); however, they are two distinct diseases and rarely occur together in the same patient [2]. Wolfram syndrome is one disorder in which DI and DM co-exist; however, it is a rare genetic disease also characterized by neurologic dysfunction including optic atrophy and deafness, and the DI in this syndrome is centrally mediated [3]. We performed an extensive search on PubMed Medline and Scopus, which returned two case reports describing diabetic ketoacidosis (DKA) complicated by central DI and one case report of a patient with type 1 DM presenting in a hypernatremic hyperosmolar state [4-6]. However, there is no literature that describes a patient with nephrogenic DI (NDI) and new-onset type 2 DM presenting in DKA.

NDI is a rare condition in which the kidneys are unable to appropriately concentrate urine in response to antidiuretic hormone (ADH) [7]. In NDI, receptors and/or channels on the collecting ducts of nephrons are defective, causing inappropriate excretion of dilute urine and an elevated serum sodium. Manifestations of the disease are polyuria and compensatory polydipsia [8]. There is no curative treatment for NDI. Management consists of dietary sodium restriction, thiazide diuretics, non-steroidal anti-inflammatory drugs, and increased water intake in response to thirst [9].

DM is a more common disease and can be complicated by DKA. The standard management of a dehydrated pediatric patient in DKA consists of isotonic saline (0.9% sodium chloride) administered as a weight-based bolus [10]. In a patient with concomitant NDI, this initial sodium load could be devastating by precipitating an acute hypernatremia. The recommended intravenous fluid replenishment for pediatric patients with NDI is dextrose 5% in water with an infusion rate that slightly exceeds the urine output [11]. However, the use of hypotonic fluids has the potential to lower serum osmolality too quickly, which can result in cerebral edema, and dextrose-containing solutions as an initial fluid for patients in DKA can exacerbate their critical hyperglycemia. This case report provides a framework for initial fluid resuscitation, both oral and intravenous, for the unique challenge of a pediatric patient with NDI who presented to the emergency department (ED) with new-onset type 2 DM in DKA.

## Case presentation

A 16-year-old male with NDI, obesity, and hypertension presented to the pediatric ED for one day of generalized weakness and decreased appetite. The patient had known NDI, diagnosed shortly after immigration to the United States several years prior.

The patient suffered from polyuria and compensatory polydipsia prior to his formal diagnosis of NDI. He was initially treated with a thiazide diuretic. However, by the time of his ED presentation, he had last seen a nephrologist three years prior and had not been adherent to his thiazide medication.

The patient reported drinking approximately 15 to 20 L of water per day. He was an employee at a fast-food restaurant and frequently ate food without attempts to limit his dietary salt intake. The patient denied alcohol use. He was not taking any medications known to cause NDI, such as lithium.

He developed profound weakness over one day and was unable to tolerate standing or walking for more than a few minutes, which prompted his presentation to the ED. He denied cough, shortness of breath, chest pain, nausea, vomiting, or diarrhea. He also denied fevers, although endorsed feeling “hot on the inside.”

Vital signs were: temperature 36.9^o^C, pulse rate 134 beats per minute, blood pressure 142/96 mmHg, respiratory rate 18 breaths per minute, and oxygen saturation 96% in ambient air. Physical examination was significant for morbid obesity, somnolence, dry mucous membranes, delayed capillary refill to four seconds, and acanthosis nigricans on his neck. The patient requested several cups of iced water throughout the interview, which we provided to him.

The first tests ordered were a chemistry panel and a point-of-care blood glucose. His point-of-care blood glucose was undetectably high, reported as “greater than 600 mg/dL” on the glucometer. Due to his level of hyperglycemia, we obtained laboratory tests for DKA (see Table 1).

**Table 1:** Initial Laboratory Values pCO_2_, partial pressure of carbon dioxide; pO_2_, partial pressure of oxygen; BUN, blood urea nitrogen.

Test result	Value	Reference range
Venous blood gas		
pH	7.13	7.33-7.43
pCO_2_	47 mmHg	41-51 mmHg
pO_2_	35 mmHg	35-45 mmHg
Bicarbonate	15.6 mmol/L	22.0-26.0 mmol/L
Lactate	3.6 mmol/L	1.0-2.4 mmol/L
Sodium	140 mmol/L	136-145 mmol/L
Potassium	6.2 mmol/L	3.5-5.3 mmol/L
Glucose	635 mg/dL	74-99 mg/dL
Chemistry panel		
Glucose	1,065 mg/dL	74-99 mg/dL
Sodium	137 mmol/L (corrected for hyperglycemia, 153 mmol/L)	136-145 mmol/L
Potassium	7.5 mmol/L (no hemolysis detected)	3.5-5.3 mmol/L
Chloride	94 mmol/L	98-107 mmol/L
Bicarbonate	13 mmol/L	18-27 mmol/L
BUN	29 mg/dL	6-23 mg/dL
Creatinine	1.58 mg/dL	0.60-1.10 mg/dL
Calcium	10.9 mg/dL	8.5-10.7 mg/dL
Phosphorus	7.2 mg/dL	3.1-5.1 mg/dL
Albumin	5.6 g/dL	3.4-5.0 g/dL
Anion gap	30 mEq/L	8-12 mEq/L
Urinalysis		
Color	Colorless	Straw, yellow
Appearance	Clear	Clear
Specific gravity	1.014	1.005-1.035
pH	5.0	5.0-8.0
Protein	100 mg/dL	Negative mg/dL
Glucose	≥500 mg/dL	Negative mg/dL
Blood	Small	Negative
Ketones	20 mg/dL	Negative mg/dL
Leukocyte	Negative	Negative
Esterase nitrite	Negative	Negative
Other relevant values		
Serum osmolality	367 mOsm/kg H_2_O	280-300 mOsm/kg H_2_O
Urine osmolality	342 mOsm/kg	200-1,200 mOsm/kg
Serum beta-hydroxybutyrate	7.75 mmol/L	0.02-0.27 mmol/L

The patient was determined to be in moderate DKA from new-onset DM. An electrocardiogram obtained due to hyperkalemia showed peaked T waves, and IV calcium gluconate was administered.

Due to the patient’s underlying NDI, extensive consultation with subspecialty services was imperative to appropriate and safe fluid resuscitation. Nephrology, endocrinology, and the pediatric intensive care unit (PICU) were notified of the patient. and a discussion was coordinated between sub-specialists to determine the appropriate fluid resuscitation. A bolus was deferred due to the risk of causing an acute hypernatremia. We started intravenous fluids (IVF) of dextrose 5% with 0.2% normal saline (D5 0.2%NS) at a rate of one-and-a-half maintenance (150 mL/hr). The patient was transferred to the PICU within 30 minutes of his diagnosis for the treatment of DKA. In the PICU, an insulin drip at 0.1 units/kg/hour was started and the IVF of D5 0.2%NS at a rate of one-and-a-half maintenance were continued. The insulin drip was not initiated in the ED per hospital protocol; however, it is important to be aware that if the patient had remained in the ED for longer the insulin drip would have been started. He was allowed to drink 250 mL of free water every one hour, rather than adhering to the nothing-by-mouth protocol that is typical of DKA management. His fluid balance goal was to be net positive, by no specific margin. Frequent laboratory monitoring included point-of-care blood glucoses and arterial blood gases every one hour, serum osmolality levels every two hours, and chemistry panels every four hours (see Table 2).

**Table 2:** Trend of Serum Chemistry Values and Fluid Intake and Output in the First 24 Hours of Treatment IVF, intravenous fluid; PO, oral; D5 0.2%NS, dextrose 5% with 0.2% normal saline. Interventions: ^a^IV calcium gluconate given; ^b^insulin regular infusion 0.05 units/kg/hr started; D5 0.2%NS at 1.5 maintenance rate started; ^c^increased insulin regular infusion to 0.1 units/kg/hr; ^d^the patient had dinner; insulin glargine and lispro injections given; insulin regular infusion stopped, one hour later; D5 0.2%NS stopped and 0.45%NS started at the time the insulin infusion was stopped.

	Time of presentation^a^	Hour 0 (start of treatment)^b^	Hour 4^c^	Hour 8	Hour 12	Hour 16^d^	Hour 20	Hour 24	Total
Glucose, mg/dL	1,096	1,092	710	463	257	232	315	315	
Sodium (corrected for hyperglycemia), mmol/L	137 (153)	137 (153)	142 (152)	144 (150)	144 (147)	141 (143)	137 (140)	136 (139)	
Potassium, mmol/L	7.5	7.2	5.2	4.8	4.4	3.9	4.1	4.7	
Chloride, mmol/L	94	94	102	105	106	104	102	101	
Bicarbonate, mmol/L	13	12	14	21	20	23	21	20	
Anion gap, mEq/L	30	31	26	18	18	14	14	15	
Serum osmolality, mOsm/kg H_2_O	367	365	358	341	323	315	314	309	
Urine osmolality, mOsm/kg	342	376		392		404	359	302	
Intake and output									
PO intake, mL		250	250	750	600	742	220	220	3,032
IVF intake, mL			580	607	631	412	611	412	3,253
Urine output, mL		1,300	700	490		560	425	725	4,200

His potassium improved after initiation of the insulin infusion. Potassium was not added to the maintenance fluids, nor were any potassium replacements required in the first 24 hours of treatment. His anion gap normalized approximately 13 hours after initiation of treatment. He was provided a low salt and low carbohydrate diet at 16 hours of treatment, and subcutaneous long-acting and short-acting insulin were administered. One hour later, the insulin drip was stopped per protocol. At the time the insulin drip was stopped, IVF were switched to 0.45%NS at maintenance rate because the patient remained mildly dehydrated and his sodium levels were stable. His oral water intake was no longer restricted. The next morning, approximately 27 hours into his treatment, IVF were discontinued because his serum sodium levels were stable and he was clinically euvolemic. His serum sodium levels were maintained in the normal range for the remainder of his hospital stay. His hemoglobin A1C returned at 8.9%, insulin level 8 μIU/mL and C-peptide 1.2 ng/mL. GAD65 antibody, insulin antibody, and islet cell antibody levels were 0. He was diagnosed with type 2 DM.

## Discussion

Our patient was diagnosed with NDI at his first visit with nephrology in the United States based on several clinical findings. As an infant, his mother reported that he preferred water to formula or breastmilk, and throughout childhood had enuresis, although no behavioral interventions were ever tried. The patient had been treated with desmopressin without improvement, despite up-titration of the dose. At an office visit, his measured early morning serum sodium was 150 mmol/L, serum osmolarity was 314 mOsm/kg H_2_O, with an inappropriately dilute urine osmolality of 89 mOsm/kg. Copeptin levels were never measured. There was no family history of DI. However, the patient’s younger brother had frequent urination, and his mother had experienced increased thirst during her pregnancies, with a few instances of fainting due to dehydration. Although no genetic test was done to confirm NDI in our patient (his mother declined testing), congenital X-linked NDI was suspected, caused by a mutation in arginine vasopressin receptor 2 gene (AVPR2) which leads to an abnormal ADH receptor. This diagnosis was based on history, labs, partial symptoms in his mother (possibly explained by a skewed X-linked inactivation, with symptoms exacerbated in times of stress, e.g., pregnancy), and symptoms in his brother. The patient’s maternal grandmother and maternal grandfather had type 2 DM.

DM is much more common than DI (globally, one in 11 adults have DM, whereas approximately one in 25,000 people have DI) [12,13]. DKA in patients with DM is a hyperglycemic emergency characterized by a laboratory triad of hyperglycemia, ketonemia, and metabolic acidosis [14,15]. Management of DKA requires frequent clinical and laboratory assessments including monitoring of vital signs, intravenous fluid intake, urinary output, electrolyte levels, and acid-base status [14].

**Figure 1:**
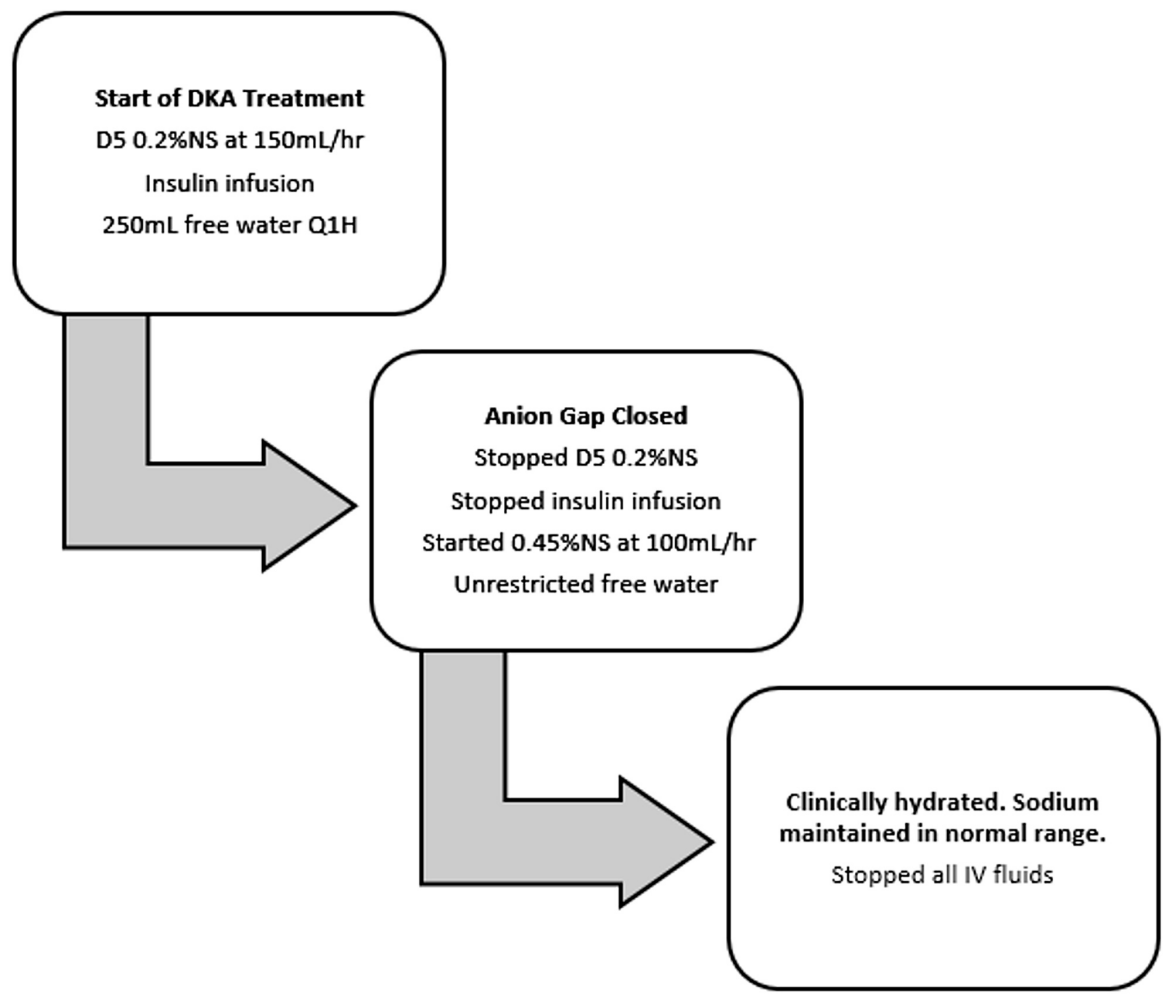
Fluid Resuscitation Guide for Patients With NDI in DKA Based on Our Management

The management of patients with NDI and DM in DKA is challenging and no guidelines exist for their initial fluid resuscitation, which is a critical component of DKA management. Providing 0.9% sodium chloride (NS), the preferred intravenous fluid in DKA treatment, as a bolus or continuous infusion, can be dangerous in patients with NDI because it can precipitate acute increases in serum sodium. Guarino et al. recommend dextrose 5% in water for intravenous resuscitation of pediatric patients with NDI, and reserving the use of NS for patients with NDI who are in shock [11]. Clinically our patient was determined not to be in shock based on his hemodynamic stability, so we deferred the use of NS. Serious consequences of hypernatremia include intracranial hemorrhage from rupture of vessels due to brain shrinkage, seizures, and death [16,17]. Dysnatremia has been found to be an independent indicator of mortality in hospitalized patients, and the mortality rate associated with hypernatremia may be as high as 20% [16,18]. On the contrary, administration of hypotonic fluids can cause overly rapid correction of hypernatremia and hyperosmolality and lead to the devastating outcome of cerebral edema [17]. In the case of our patient, thoughtful management with sub-specialist consultation was of paramount importance in safe and timely volume resuscitation. We chose to allow free water oral intake in addition to IVF with D5 0.2%NS to provide intravascular repletion without precipitating rapid drops in serum osmolality. A rate of 1.5 to 2 times maintenance was recommended by nephrology to restore intravascular volume and improve his acute renal injury. We chose 0.2%NS because it would decrease his serum sodium by approximately 0.3 mmol/L/hr at 150 mL/hr, as opposed to NS which would not be expected to decrease his serum sodium. This rate is estimated by the Adrogue-Madias formula for sodium correction. 0.45% NS was also considered; however, we chose 0.2%NS to ensure a decrease in osmolality with a reasonable infusion rate of 150 mL/hr versus upwards of 200 mL/hr for a higher tonicity fluid to achieve the same decrease in serum sodium. We added dextrose to avoid decreasing osmolality too quickly, keeping in mind that frequent laboratory monitoring would allow titration of fluids as needed. We started 0.45%NS at maintenance rate after the insulin drip was discontinued and liberalized the patient’s free water intake. Due to the patient’s acute kidney injury, this higher sodium content was deemed appropriate to improve the intravascular volume and renal perfusion, with the understanding that his sodium levels may increase as his acute kidney injury improves due to increased solute delivery to the distal nephron and further exacerbation of his NDI. His acute kidney injury resolved with sodium levels remaining within normal limits. IVF were safely discontinued when the patient was intravascularly euvolemic and able to respond to an intact thirst drive. Despite this patient's complexity, he experienced a good outcome due to timely multidisciplinary assessment and an organized management strategy. This management plan provides a framework that may be helpful to physicians who are faced with similar cases (see Figure 1). 

## Conclusions

The co-existence of NDI and DM in DKA in a patient is rare. Choice of initial fluid resuscitation must be done thoughtfully but with urgency, taking care to balance the risk of worsening hypernatremia with the risk of decreasing osmolality too quickly. This case report provides necessary guidance to emergency medicine, intensive care, and inpatient physicians on fluid resuscitation in dehydrated patients with co-existent NDI and DM in DKA, which has previously never been described.
